# Functional annotation of 19,841 *Populus nigra *full-length enriched cDNA clones

**DOI:** 10.1186/1471-2164-8-448

**Published:** 2007-12-03

**Authors:** Tokihiko Nanjo, Tetsuya Sakurai, Yasushi Totoki, Atsushi Toyoda, Mitsuru Nishiguchi, Tomoyuki Kado, Tomohiro Igasaki, Norihiro Futamura, Motoaki Seki, Yoshiyuki Sakaki, Kazuo Shinozaki, Kenji Shinohara

**Affiliations:** 1 Department of Molecular and Cell Biology, Forestry and Forest Products Research Institute (FFPRI), 1 Matsunosato, Tsukuba, Ibaraki 305-8687 JAPAN; 2 RIKEN Plant Science Center, 1-7-22, Suehiro-cho, Tsurumi-ku, Yokohama, Kanagawa 230-0045 JAPAN; 3 RIKEN Genomic Science Center, 1-7-22, Suehiro-cho, Tsurumi-ku, Yokohama, Kanagawa 230-0045 JAPAN; 4 Hayama Center for Advanced Studies (HCAS), The Graduate University for Advanced Studies, Shonan Kokusai-mura, Hayama-cho, Miura, Kanagawa 240-0193 JAPAN

## Abstract

**Background:**

*Populus *is one of favorable model plants because of its small genome. Structural genomics of *Populus *has reached a breakpoint as nucleotides of the entire genome have been determined. Reaching the post genome era, functional genomics of *Populus *is getting more important for well-comprehended plant science. Development of bioresorce serving functional genomics is making rapid progress. Huge efforts have achieved deposits of expressed sequence tags (ESTs) in various plant species consequently accelerating functional analysis of genes. ESTs from full-length cDNA clones are especially powerful for accurate molecular annotation. We promoted collection and annotation of the ESTs from *Populus *full-length enriched cDNA clones as part of functional genomics of tree species.

**Results:**

We have been collecting the full-length enriched cDNA of the female poplar (*Populus nigra *var. *italica*) for years. By sequencing *P. nigra *full-length (PnFL) cDNA libraries, we generated about 116,000 5'-end or 3'-end ESTs corresponding to 19,841 nonredundant PnFL clones. Population of PnFL cDNA clones represents 44% of the predicted genes in the *Populus *genome.

**Conclusion:**

Our resource of *P. nigra *full-length enriched clones is expected to provide valuable tools to gain further insight into genome annotation and functional genomics in *Populus*.

## Background

The use of forest trees as a sustainable environmental resource has underscored the importance of genomics in aiding the genetic modification of trees for preferable performance and the development of DNA markers for selective breeding. In this context, the development of the genomic resources of *Populus *has gained increasing importance because these species have a small genome (~480 Mbp) when compared to other tree species. For example, international groups of researchers have determined the nucleotide sequence of the entire genome of the black cottonwood (*Populus trichocarpa*) [1,2]. But because the sexual reproduction span of *Populus *is long, it has proven to be an unsuitable model for forward genetics like mutant-based studies. Reverse genetic approaches based on the functional genomics are therefore essential. As one of important tools, *Populus *ESTs have been collected [3-13]. The publicly available EST collections of *Populus *including poplar, aspen, cottonwood and their hybrids have already grown to 385,000 [13].

Full-length cDNA resources are very useful, not only for gene annotation and the determination of transcriptional start sites, but also for functional analyses [14], especially when analyzed within the context of genomic sequences. Various methods have been developed that allow preferential cloning of cDNA that corresponds to full-length mRNAs that have 5'-proximal cap structures [15]. These methods have been applied to large-scale analyses of transcripts from human [16], mouse [17,18], fruit fly [19], rice [20], *Arabidopsis *[21], and moss [14]. Although population of *Populus *EST has grown steadily, the ESTs may appear not to be from full-length cDNAs to a large extent. Recently, *Populus *ETS has been tried to obtain from a full-length enriched cDNA library constructed by the method of the oligo-capping [22] or the biotinylated CAP trapper [2,13]. In the study we report herein, we constructed a full-length enriched cDNA library from poplar (*Populus nigra *var. *italica*) by using the biotinylated CAP-trapper method.

Functional annotation of ESTs that uses integrated prediction tools and proper curation of the results is not only necessary to complete the annotation process but to find actual biological processes. We annotated our *P. nigra *ESTs primarily by using the BLAST program to search the databases of The Institute for Genomic Research (TIGR) [23] and The Universal Protein Resource (UniProt) [24]. Although 90% of our PnFL clones were identified through these databases, the rest remained functionally unknown. To identify the remaining PnFL clones, we substituted the coding sequences (CDS) of *P. trichocarpa *for PnFL ESTs. 65% of the substituted CDS was able to be described using the BLAST against the public protein databases. We treated 35% rest with another computational work of a protein domain analysis. Resultant domains found in these sequences may provide a critical clue to understand molecules specific in trees and/or *P. nigra*, a series of such substitutive procedures is somewhat artificial though. Furthermore genome-wide analysis of poplar was done using comparative genomics with herbaceous model plants *Arabidopsis *and rice in the present study. We anticipate that the information we gathered in this comparative analysis will make possible global comparisons among plant species by using functional genomics.

## Results and Discussion

### Quality of the cDNA Library

We constructed a full-length enriched cDNA library (PnFL2) from *P. nigra *buds, roots, twigs, and stress-treated leaves by using the biotinylated CAP-trapper method, together with trehalose-thermoactivated reverse transcriptase [25]. This library was named PnFL2 after *P*. *nigra *full-length cDNA library version 2. The quality of the cDNA library was evaluated before large-scale sequencing by examining 96 randomly selected but representative clones. The mean size of the insert DNA was estimated to be about 1.4 kb (range, 0.8 kb to 3.5 kb) by measuring the length of the *Pvu*II fragments of 86 independent clones from among the 96 sample clones.

**Figure 1:**
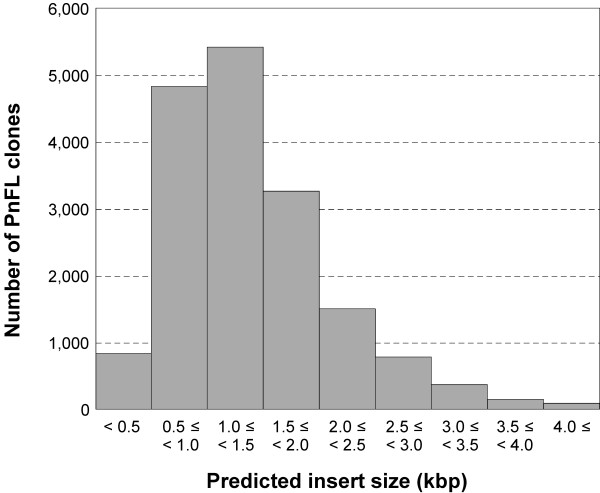
Distribution of the predicted insert size in the second version of the *P. nigra *full-length cDNA library (PnFL2). The fragment sizes of 17,273 *P. trichocarpa *CDS that were substituted for the *P. nigra *ESTs were determined.

To further estimate the distribution of the insert sizes of the PnFL2 clones, we created a histogram showing a length distribution of the *P. trichocarpa *CDS that were substituted for the *P. nigra *ESTs. 18,578 PnFL2 nonredundant clones were corresponded to *P. trichocarpa*'s predicted CDS under conditions as following: 1) both the 5'-end and 3'-end sequences of each PnFL2 clone had to have blastn hit against the *P. trichocarpa *CDS in their proper orientation; 2) *E *value of the blastn hit had to be less than 1e-35 of both the 5'-end and 3'-end sequences; 3) *E *value of the hit had to be less than 1e-50 of the 5'-end or 3'-end sequences. Altogether, 17,273 PnFL2 clones satisfied all conditions above. And 17,273 corresponding *P. trichocarpa *CDS were defined as the substituted CDS and used for the histogram. The length of most of the 17, 273 substituted CDS ranged between 1.0 kbp and 1.5 kbp (Fig. 1).

By comparing the transcripts longer than 3.0 kbp with the ESTs contained in the PnFL2 library, we found 631 corresponding substituted CDS. This computational estimation was partially reconfirmed by subjecting 96 of the extracted 3.0-kbp or greater sized clones to electrophoresis (data not shown). We found that the PnFL population would provide useful resources for *Populus *researchers with well intact molecules.

To analyze the cDNA population, on the other hand, 96 selected clones were sequenced from their 5'-ends and blastn-searched in the GenBank nucleotide database, resulting that 92.7% of clones contained an insert. Clones whose query start position was greater than the hit start position in the aligned region were defined as being full-length. The ratio of full-length clones was calculated as A/(A+B), wherein A is the number of full-length clones and B the number of those that are shorter. This calculation yielded a ratio of 0.75. Overall, the duplication rate of the genes in the PnFL2 library was substantially lower than that of the PnFL1 library [22], most likely because of the normalization process used in the construction of the PnFL2 library.

### One-pass sequencing of PnFL2 clones and integrated clustering of PnFL ESTs

We randomly selected 39,936 clones (PnFL2-001_A01 through PnFL2-104_P24) from the PnFL2 cDNA library and sequenced them from the 5'-end and the 3' end by using a high-throughput DNA sequencing process. We identified 39,183 clones that had 5'-end-based and/or 3'-end-based ESTs (phred quality value of ≥ 20). The nucleotide sequences of the PnFL2 ESTs have been submitted to the DDBJ/EMBL/GenBank [DB874873 through DB910976] and are provided in Additional file 1. These ESTs were first clustered by using the phrap program, and the phrap-assembled sequences were then subjected to a round-robin blastn search within their own population. Through these clustering processes, we obtained 15,581 tentative contigs and 17,412 singletons that did not have a partner with pairwise homology either within the total pool of sequences or within a given cluster, representing 18,578 nonredundant PnFL2 clones. We also found that the consensus sequences of 2,387 tentative contigs and of 3,421 singletons each covered a whole transcript. For better annotation of *Populus *genes and comparative studies among plant species, complete reading of full-length clones should be important. Although our present work did not focus only on completely read cDNAs, we obtained those of 5,808. Other group also collected 4,664 *Populus *cDNAs whose sequences were completely read [2]. Altogether, this information should be valuable in the functional annotation of the *Populus *genome.

When the 4,522 PnFL1 ESTs [22] were filtered out, 4,316 PnFL2 ESTs remained. These remaining ESTs were integrated and assembled into clusters as described in the Methods section. For each cluster, we considered the clone that had the longest 5' read among other cluster members to be a cluster representative. This procedure allowed us to eliminate diverse variations in the length of the 5'-untranslated region within each cluster. The resulting population of nonredundant PnFL clones was comprised of cluster representatives and singletons. In total, 19,841 nonredundant PnFL clones were obtained; 17,153 were PnFL2-specific, 1,263 were PnFL1-specific, and 1,425 were common to both the PnFL2 and PnFL1 libraries (PnFL2/PnFL1-common). This population of nonredundant clones corresponds to 44% of the entire *Populus *CDS [13].

### Functional annotation and classification of PnFL ESTs

**Figure 2:**
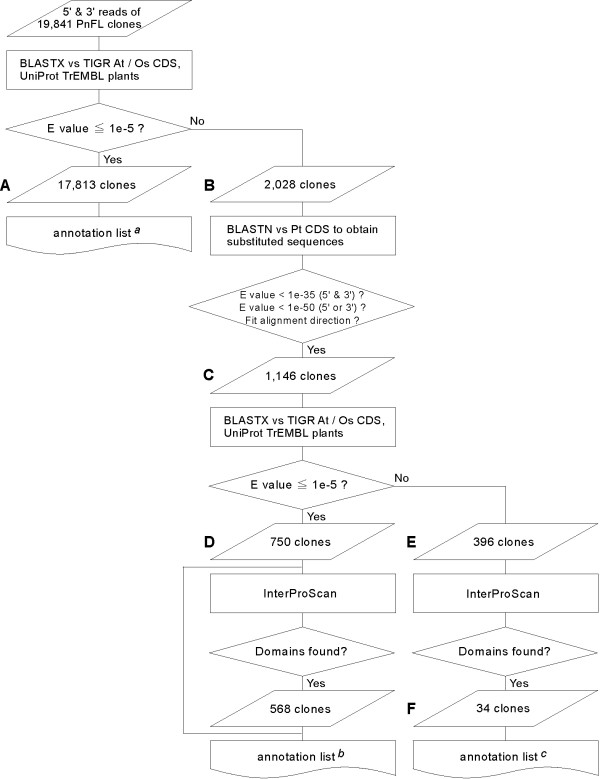
Flow chart of the functional annotation of PnFL cDNA clones. In total, 19,841 nonredundant PnFL clones were subjected to functional annotation. Parallelogrammatic elements with a left number indicate the results of each adjacent procedure (see *Results and discussion*). The annotation lists are summarized in ^a^Additional file 2, ^b^Additional file 3 and ^c^Table 1.

We tried to delimit the features of the proteins that the PnFL clones encoded as illustrated in Figure 2. Both the 5' and 3' reads were first annotated based on searches for homologous sequences in the TIGR and UniProt public databases by using the blastx program with each PnFL EST as a query against the *Arabidopsis *CDS in the TIGR *Arabidopsis thaliana *Database [26], the rice CDS in the TIGR Rice Genome Annotation database [27], and the UniProt TrEMBL plant protein database [24]. Currently, these databases are the authoritative resources for plant protein sequences and functional information.

In these databases searches, 17,813 PnFL clones hit with an *E *value of ≤ 1.0e-5 (Fig. 2A; Additional file 2). The remaining 2,028 clones that did not hit (Fig. 2B) were converted into *P. trichocarpa *CDS, as described above, yielding 1,146 substituted CDS (Fig. 2C). The three public databases were searched again to determine whether these substituted sequences were homologous to any published sequences. In a separate search performed with the InterProScan program [28], 750 substituted clones hit with an *E *value of ≤ 1.0e-5 (Fig. 2D; Additional file 3), and the remaining 396 clones (Fig. 2E) that did not hit are possible candidates for tree-specific (or *P. nigra*-specific?) genes or unhopefully genes due to contaminations of total RNAs. Using the same InterProScan program, we defined a protein feature of these 396 clones; any domains were found in only 34 clones (Fig. 2F; Table 1). Although this information was derived from artificially substituted CDS, descriptions of these hard-to-annotate clones will allow us to interpret the unique nature of *Populus *species.

**Table 1:** Domain detection by InterProScan for characterizing of no-hit clones ^a^

Clone name ^b^	Accession	Name	*E *value
PnFL1-083_I10	IPR012336	Thioredoxin-like fold	0.0069
PnFL2-001_C15	IPR000480	Glutelin	5.40E-06
PnFL2-003_H05	IPR001810	Cyclin-like F-box	0.0014
PnFL2-004_G12	IPR007836	Ribosomal protein L41	1.60E-08
PnFL2-009_G19	IPR006031	XYPPX repeat	19
PnFL2-009_I06	IPR003882	Pistil-specific extensin-like protein	1.60E-07
PnFL2-010_B02	IPR006031	XYPPX repeat	64
PnFL2-010_P09	IPR006121	Heavy metal transport/detoxification protein	1.00E-09
PnFL2-013_P01	IPR000772	Ricin B lectin	2.20E-23
	IPR008997	Ricin B-related lectin	1.40E-30
PnFL2-016_G15	IPR010978	tRNA-binding arm	0.0046
PnFL2-021_F12	IPR000480	Glutelin	9.90E-05
PnFL2-021_J01	IPR000167	Dehydrin	0.00011
PnFL2-026_J14	IPR001627	Semaphorin/CD100 antigen	9.042
PnFL2-028_L04	IPR000480	Glutelin	9.90E-05
PnFL2-032_H01	IPR000048	IQ calmodulin-binding region	7.401
PnFL2-034_F13	IPR000480	Glutelin	6.50E-07
	IPR000976	Wilm's tumour protein	9.60E-05
	IPR006706	Extensin-like region	1.20E-31
PnFL2-034_J01	IPR010978	tRNA-binding arm	0.0046
PnFL2-036_J14	IPR001810	Cyclin-like F-box	5.50E-05
PnFL2-046_A21	IPR000772	Ricin B lectin	2.20E-23
	IPR008997	Ricin B-related lectin	1.40E-30
PnFL2-046_B04	IPR000480	Glutelin	4.50E-07
PnFL2-048_D17	IPR006031	XYPPX repeat	64
PnFL2-048_H02	IPR000048	IQ calmodulin-binding region	7.401
PnFL2-055_F11	IPR003267	Small proline-rich	3.10E-05
PnFL2-064_O17	IPR000480	Glutelin	1.90E-05
	IPR003882	Pistil-specific extensin-like protein	5.00E-06
PnFL2-067_N14	IPR009424	Protein of unknown function DUF1070	1.10E-27
PnFL2-075_P11	IPR001810	Cyclin-like F-box	5.90E-05
PnFL2-076_H24	IPR006031	XYPPX repeat	19
PnFL2-077_G19	IPR001878	Zinc finger, CCHC-type	1.70E-06
PnFL2-078_N14	IPR001179	Peptidylprolyl isomerase, FKBP-type	0.00067
PnFL2-079_I20	IPR006077	Vinculin/alpha-catenin	2.40E-05
PnFL2-087_M22	IPR000048	IQ calmodulin-binding region	7.401
PnFL2-090_P08	IPR008011	Complex 1 LYR protein	3.70E-15
PnFL2-098_J19	IPR002885	Pentatricopeptide repeat	2.80E-08
PnFL2-102_L04	IPR000480	Glutelin	1.30E-06
	IPR003882	Pistil-specific extensin-like protein	1.90E-07

^a ^Substituted *P. trichocarpa *CDS for PnFL ESTs were subjected to the InterProScan program. This table corresponds to 'annotation list ^c ^' in Fig. 2.

^b ^PnFL clones whose substitutive sequences have at least one hit are listed.

**Figure 3:**
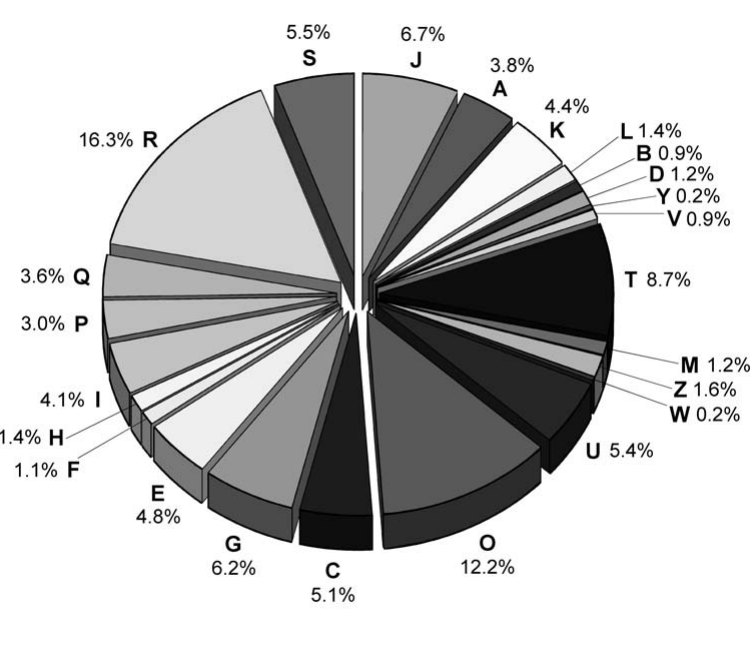
Overview of the functional classification of the *P. nigra *ESTs. In total, 10,829 of the 19,841 nonredundant ESTs that comprised the 5' or 3' reads that yielded the lowest *E *value for each clone were assigned to eukaryotic clusters of KOGs. Designations of functional categories and the proportion of each: A, RNA processing and modification; B, chromatin structure and dynamics; C, energy production and conversion; D, cell cycle control and mitosis; E, amino acid transport and metabolism; F, nucleotide transport and metabolism; G, carbohydrate transport and metabolism; H, coenzyme transport and metabolism; I, lipid transport and metabolism; J, translation, ribosomal structure, and biogenesis; K, transcription; L, replication and repair; M, cell wall/membrane/envelope biogenesis; O, posttranslational modification, protein turnover, and chaperone functions; P, inorganic ion transport and metabolism; Q, secondary metabolite biosynthesis, transport, and catabolism; T, signal transduction; U, intracellular trafficking, secretion, and vesicular transport; V, defense mechanisms; W, extracellular structures; Y, nuclear structure; Z, cytoskeleton; R, general functional prediction only; and S, function unknown.

Figure 3 shows the functional classification of the putative proteins encoded by the *P. nigra *ESTs on the basis of their assignment to eukaryotic clusters of orthologous groups of proteins (KOGs). KOGs includes proteins from 7 eukaryotic genomes: 3 animals (*Caenorhabditis elegans*, *Drosophila Melanogaster*, and *Homo sapiens*), one plant (*A. thaliana*), two fungi (*Saccharomyces cerevisiae *and *Saccharomyces pombe*), and an intracellular microsporidian parasite (*Encephalitozoon cuniculi*) [29,30]. Of the 19,841 putative PnFL proteins derived from either the 5' read or 3' read, 10,829 (54.6%) were assigned to KOGs by using the blastx program (*E *< 1.0e-10) and subsequent emulation of the sequences as described previously [22]. The rate assigned to the KOGs of the integrated PnFL ESTs was higher than that of the stress-related *P. nigra *ESTs (45%), probably because the new PnFL2 cDNA library was generated by using RNAs from various organs of *P. nigra *together with longer reads of the PnFL2 clones. The proportion of items for the classification seemed to be similar between the two libraries as a whole.

### Comparative genomic analysis of PnFL ESTs

**Figure 4:**
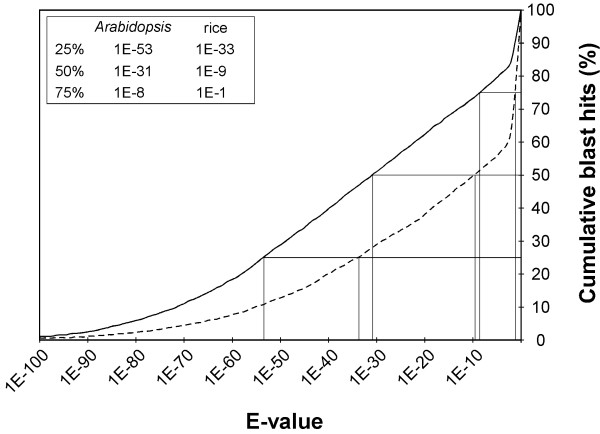
Cumulative count of homologs of *Arabidopsis *and rice. All the CDS of both *Arabidopsis *and rice were compared with the PnFL ESTs by using the tblastx program. The curves depict the percentages of genes in the *Arabidopsis *(solid line) and the rice (broken line) genomes that have greater sequence similarity than the *E *value ascribed to a corresponding sequence in the PnFL ESTs. For instance, as shown in the inset, 50% of the genes have a hit with an *E *value of < 10^-31 ^in *Arabidopsis *and of < 10^-9 ^in rice.

We compared the PnFL ESTs with an entire set of genes both in *Arabidopsis *and in rice by using the tblastx program as described previously [12]. Because *Populus *species are dicotyledonous, the *E *values derived from the comparison with *Arabidopsis *were considerably lower than from the comparison with rice. Half of all the predicted proteins of *Arabidopsis *and those of rice matched with respective *E *values of < 10^-31 ^and < 10^-9 ^(Fig. 4). These results also showed that most *Arabidopsis *and rice genes share a homolog with the PnFL clones to a large extent. Consequently, such genome-wide comparative analysis of functional sequences is a powerful tool for achieving a comprehensive understanding of genetic homology among plant species.

### Putative physical mapping of PnFL ESTs

**Figure 5:**
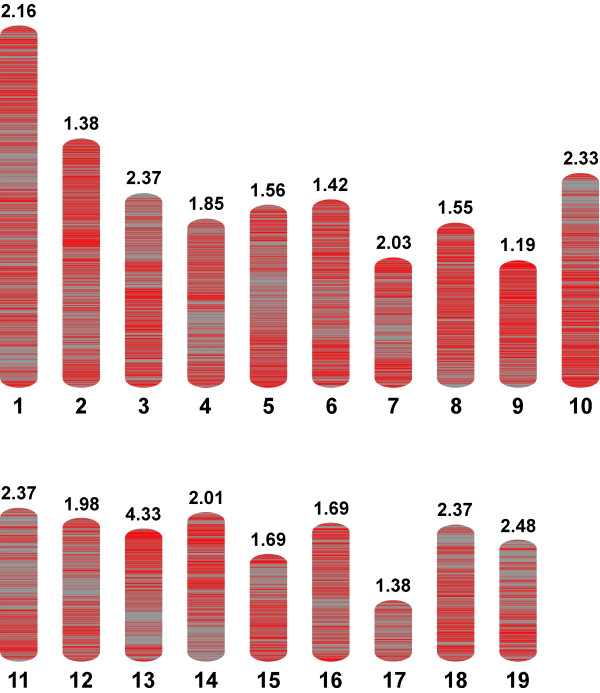
The putative physical distribution of PnFL clones in 19 *P. trichocarpa *chromosomes (top: north; bottom: south). Each red pixel shows a locus that corresponds to a PnFL clone. The number underneath each chromosome is the chromosome number and that above is the distribution index (see *Methods*).

For an overview of the distribution of our PnFL clones on the *Populus *genome, our ESTs were mimically assigned to the *P. trichocarpa *genome, whose sequences were kindly distributed by the United States Department of Energy Joint Genome Institute. The tentative genome assignment of the PnFL clones is shown as a physical map of *P. trichocarpa *chromosomes (Fig. 5). This map indicates that our PnFL clones may broadly come from all chromosomes and be distributed on each chromosome without a significant bias (the distribution index was < 2.5, except for chromosome 13).

## Conclusion

Full-length cDNAs are essential for the correct annotation of genomic sequences and for the functional analysis of genes and their products. Collections of full-length cDNAs are available in some plant species such as *Arabidopsis*, rice, moss and poplar [22]. In poplar, our collection of PnFL cDNAs was updated with 19,841 nonredundant clones. This population represented 44% of the predicted genes in the *Populus *genome. To improve the curation and distribution of this bioresource, all the PnFL cDNA clones and their applicable information will be released through RIKEN [31].

## Methods

### Plant materials and stress treatments

Leaf buds, flower buds, and portions of wooden twigs were sampled from a mature stand of female poplar (*P. nigra *var. *italica*). The samples were briefly washed with distilled water and then stored at -80°C until the RNA preparation procedure.

Explanted shoots of female poplar were axenically grown in Biopots (Watanabe TAI Co., Ltd, Osaka, Japan) that were 90 mm in diameter × 130 mm in height. The explants were covered with 100 mL of medium (pH 5.7) that contained 1× McCown's woody plant basal salt mixture (Sigma-Aldrich Corp., St. Louis, MO), 2% (w/v) sucrose, and 2% (w/v) activated charcoal that was solidified with 0.3% (w/v) gellan gum (Gelrite, Wako Pure Chemicals, Osaka, Japan). The temperature of the cleanroom was maintained at 25 ± 1°C, and cool-white fluorescent bulbs supplied 40–60 μmol m^-2 ^s^-1 ^of light alternated with 8 h of darkness.

After the plants had grown in the Biopots for ~2 months, the roots were sampled and washed with distilled water. Leaflets were then cut off at a petiole and subjected to a series of stress treatments that included dehydration, chilling, heating and exposure to NaCl, abscisic acid, salicylic acid, jasmonate, and H_2_O_2 _for varying periods (1, 2, 5, 10, and 24 h; except, 6 h for the salicylic acid and jasmonate exposure). For the dehydration treatment, the leaves were desiccated in 90 mm × 20 mm Petri dishes under dim light at 25°C and in an atmosphere of 50% to 60% humidity. For the chilling and heating treatments, the leaves were placed in Petri dishes with a wet paper towel and then exposed respectively to temperatures of 4°C and 34°C in the dark. For all other treatments, the leaves were soaked in 50-mL aqueous solutions of 400 mm NaCl, 100 μm abscisic acid, 100 μm salicylic acid, 100 μm jasmonate, and 200 mm H_2_O_2_, under dim light at 25°C. For the wounding treatment, leaflets were wounded by boring with a prick. All the samples were then frozen in liquid nitrogen for RNA extraction.

### RNA isolation and construction of full-length enriched cDNA library

Total RNA was extracted in a solution of phenol-guanidine isothiocyanate (TRIzol^® ^Reagent, Invitrogen™ Life Technologies, Carlsbad, CA). A total RNA mixture derived from all the samples described above was further purified using a MACS mRNA Isolation Kit (Miltenyi Biotec, Gladbach, Germany), and resultant poly(A)^+ ^RNA served the following construction of a cDNA library. A full length-enriched cDNA library (PnFL2 library) was constructed with a normalization step according to the method of biotinylated CAP trapper using trehalose-thermoactivated reverse transcriptase [25].

### EST sequencing and clustering

The appropriate aliquots of library solution in storage tubes were spread on Luria-Bertani (LB) agar plates containing 100 μg/mL of ampicillin and incubated at 37°C overnight.

The colonies that grew were randomly picked and inoculated into 384-well microtiter plates, the wells of which were filled with 40 μL of LB medium that contained 100 μg/mL of ampicillin. Sequencing templates were prepared from these arrayed clones by using the TempliPhi DNA Sequencing Template Amplification Kit (Amersham Biosciences, Uppsala, Sweden). The sequencing reactions were performed according to the protocol of the BigDye Terminator v3.1 Cycle Sequencing Kit (Applied Biosystems, Foster City, CA). The sequence primer used for 5' sequencing was 5'-TGT AAA ACG ACG GCC AGT-3' ; and the primers used for 3' sequencing were 5'-AGC GGA TAA CAA TTT CAC ACA GGA-3' for 16 plates that correspond to PnFL2 clones 1–16 and 5'-AAT ACG ACT CAC TAT AGG G-3' for 88 plates that correspond to clones PnFL2 17–104. The products of the sequencing reaction were purified by precipitation with ethyl alcohol and then loaded onto an Applied Biosystems ABI3700 DNA sequencer.

Raw sequence data were processed and base-called by using phred (CodonCode Corp., Dedham, MA), a base-calling program. The PnFL2 EST sequences were uploaded into a filtering program to remove vector, adaptor, and low-quality bases. These processed PnFL2 sequences were assembled with the phrap program (Laboratory of Phil Green, Genome Sciences Department, University of Washington) [32] under default conditions, except for the following criteria: -minmatch 400, -minscore 400, -repeat stringency .999, -trim quality 20. phrap-treated sequences, including tentative contigs and singletons, were sequentially processed as follows: a base with a quality value < 20 was temporarily converted into an "n"; sequences were sequentially deleted from both ends until there were at least 30 successive non-"n") bases (A, T, G or C); clones with sequences < 200 bp in length were discarded; an "n" conversion was undone and converted into the original base (A, T, G or C); and poly A/T sequences were deleted. These representative sequences were then subjected to a round-robin blastn search within their own population. In this process, sequences sharing not less than 99% identity over 200 or more contiguous bases were grouped into clusters. All the clusters were rearranged by taking the clone ID into account, that is, sequences that shared an identical clone ID were placed into the same cluster. Clones that had only one usable sequence out of the 5' and the 3' reads but had any hit were also placed into clusters.

Meanwhile, the base-called 5' and/or 3' sequences of the 4,522 nonredundant PnFL1 clones [22] were also processed as described above. The 2,489 sequences that resulted from the processing of the PnFL1 ESTs were integrated into the PnFL2 clusters by performing a blastn search under the same conditions as the round-robin blastn search for the PnFL2 clustering. The PnFL1 clones that were not integrated into the PnFL2 clusters were clustered within the remaining PnFL1 population by performing a blastn search under the conditions described above. Other removed sequences were determined on the basis of a blastn homology search against the integrated ESTs (*E *< 1.0e-50) with ribosomal RNA and organelle DNA query sequences (DDBJ/EMBL/GenBank accession: AF174629, AF206999, AF479118, AJ006440, AP000423, X52322, Y08501, Y08502, AF162215, AF168884, AF274652).

### Tentative genome assignment of PnFL clones

The 5' and 3' sequences of 19,841 nonredundant PnFL clones were aligned with the *P. trichocarpa *genome sequence by using the blastn program. The maximum and minimum hit positions that came from queries of each PnFL clone were considered to show, respectively, the southernmost and the northernmost point of each locus on a *P. trichocarpa *chromosome. The northernmost (minimum) point was assigned to the chromosome as a corresponding locus. The information derived from the 5' ESTs was adopted only in the following cases: 1) the hit chromosome numbers differed between the 5' and 3' ESTs of each PnFL clone; and 2) the difference between the maximum and minimum hit positions was over 50,000. The lengths of the intergenic regions were then calculated by using the positional information of these tentative assignments. Variance in the lengths of the intergenic regions can be used as an indicator of gene distribution within the *Populus *genome. Consequently, we used a ratio of actual variance data to data that assumed the uniform distribution of genes on chromosome: *I *= *Var*_*Data*_/*Var*_*Uni*_, wherein *I *is the distribution index. To obtain an estimate of *Var*_*Uni*_, we repeated the simulation 10,000 times and calculated an average. The intergenic regions that were < 1000 bp in length were discarded before the distribution was calculated.

## List of abbreviations

PnFL, *P. nigra *full-length; CDS, coding sequence; EST, expressed sequence tag; KOGs, orthologous groups of proteins; TIGR, The Institute for Genome Research; UniProt, The Universal Protein Resource

## Authors' contributions

TN led the design of the study, grew sample trees, performed the stress treatment and the RNA preparation, managed the construction of full-length cDNA libraries, compiled the data and drafted the manuscript. TS led the design of the study and edited the data. YT and AT did the sequencing work and the gene clustering. MN and TI helped to prepare total RNAs. TK carried out the statistical analysis of the data. NF, MS, YS, KaS and KeS participated in the design and coordination of the study. All authors read and approved the final manuscript.

## Supplementary Material

Additional file 1 List of DDBJ accession numbers in dbEST for the full-length enriched ESTs of *P. nigra* Click here for file

Additional file 2 BLASTX analysis of the 17,813 PnFL non-redundant ESTs against AtCDS, OsCDS and the Uniprot TrEMBL plant protein database. Click here for file

Additional file 3 BLASTX analysis and InterProScan of the 750 substituted *Populus *CDS Click here for file
